# The Arachidonic Acid Metabolism Mechanism Based on UPLC-MS/MS Metabolomics in Recurrent Spontaneous Abortion Rats

**DOI:** 10.3389/fendo.2021.652807

**Published:** 2021-04-02

**Authors:** Meihe Li, Yang Haixia, Minchao Kang, Peng An, Xili Wu, Huimin Dang, Xin Xu

**Affiliations:** ^1^ Beijing Traditional Chinese Medicine Hospital Affiliated to Capital Medical University, Beijing, China; ^2^ Department of Traditional Chinese Medicine, Second Affiliated Hospital of Xi’an Jiaotong University, Xi’an, China; ^3^ Health Science Center of Xi’an Jiaotong University, Xi’an, China; ^4^ Department of Gynaecology, Beijing Traditional Chinese Medicine Hospital Affiliated to Capital Medical University, Beijing, China

**Keywords:** recurrent spontaneous abortion, metabolomics, arachidonic acid metabolism, mechanism, prostaglandin

## Abstract

Recurrent spontaneous abortion (RSA) remains a critical and challenging problem in reproduction. To discover novel biomarkers for RSA, ultra performance liquid chromatography/tandem mass spectrometry (UPLC-MS/MS) metabolomics approach was applied to detect RSA serum metabolic profiles and explore its possible pathogenesis and mechanism. The abortion rat model was established, and a metabolomics analysis was performed to evaluate the differentially expressed metabolites between the control and model groups. Immunohistochemistry (IHC), qRT-PCR, and Western blot further examined the expression of Arachidonic acid metabolism-related genes in uterus tissues. To identify arachidonic acid metabolism-related changes in RSA, ELISA’s potential mechanisms were further confirmed in serum. Ninety-one metabolites were significantly different between the two groups, as indicated by a VIP ≥1, fold change ≥1. The metabolic pathways involving arachidonic acid metabolism pathway (*P* = 0.00044) are related to RSA. Verification by experimental showed that compared with the control rats, the expression of the COX-1, COX-2, PTGFR, and TBXA2R genes associated with the arachidonic acid metabolism pathway has significantly increased the uterus and serum of RSA rats (*P* < 0.05). Regulation of the arachidonic acid metabolism pathway might serve as a promising therapeutic strategy for relieving RSA women’s symptoms.

## Introduction

Natural abortion occurs in 10% of women with normal pregnancy, and recurrent spontaneous abortion (RSA) accounts for about 5% (1). Many reasons are leading to recurrent abortion, among which endocrine dysfunction (2–4), immunity (5, 6), genetics (7, 8), infection (9), and anatomical abnormalities (10–13) are the main risk factors for recurrent abortion. Studies (14) have found that women who have had an abortion experience have a risk of up to 75% in the second pregnancy, which seriously affects patients’ physical and mental health. However, approximately 50% of RSA causes remain unknown. Therefore, exploring the causes of RSA is one of the necessary and urgent problems.

Human normal physiological activities will produce specific metabolites, but the metabolism may damage maternal and fetal health in some pregnant women (15). To complete normal pregnancy, the health of the mother’s womb and pregnancy-related disease research is essential. Therefore, we explore the effects of metabolites in the participation in the process of pregnancy. However, there is a lack of systematic study on serum metabolites and markers of recurrent abortion. In the post-genomic era, when changes in protein expression and transcription are challenging to measure, metabolomics can make up for the deficiency of proteomics. Many metabolites are downstream products of the genome and can respond to biological systems and detect changes in metabolites by amplifying biological information (16). However, metabolomics has rarely been used to identify novel metabolic biomarkers for RSA, and since the specific pathogenesis of RSA remains unclear, this valuable tool can be used as a means to reveal its underlying etiology.

Therefore, in this study, first of all, to establish the rat model of RSA studied in this experiment, based on the research of RSA in the rats and the control group of normal pregnancy rats’ serum metabolic differences, and identified and validated through the experiment the RSA possible metabolic biomarkers and disease development may be involved in signaling pathways, to develop potential targets and treatment way of treatment for RSA.

## Materials and Methods

### Animals and Ethics Statement

A total of 16 female Lewis rats (10–12 weeks) and 8 male Lewis rats were obtained from the Experimental Animal Center of Xi’an Jiaotong University (Xi’an, China) and housed in a standardized environment: temperature, 22°C; 50% to 60% humidity, and 12:12 h light–dark cycle with access to laboratory rodent chow and tap water ad libitum under pathogen-free conditions in the Experimental Animal Center of Xi’an Jiaotong University (Xi’an, China). The animal study was reviewed and approved by the ethic committee of the First Affiliated Hospital of Xi’an Jiaotong University.

### Rat Recurrent Spontaneous Abortion Model Establishment

The rats were randomly divided into two groups. Model group: a total of eight rats were used to establish the abortion model, were given hydroxyurea solution (hydroxyurea powder dissolved in normal saline at a concentration of 450mg/kg/d) intragastric administration for 10 days, followed by subcutaneous injection of 0.3mg/kg/d of epinephrine on the fourth day for 7 days. Control group: a total of eight healthy non-pregnant rats were enrolled, were given normal saline intragastricly and subcutaneously, with the same time and method as the model group. On the 11th day, male and female rats mated at 1:2; if vaginal suppositories or sperm were found, it was considered the first day of pregnancy. At 9:00 am on the third day of pregnancy, head and neck subcutaneous injection mifepristone solution 5 mg/kg once (mifepristone grind powder after dissolved in anhydrous ethanol, then suspended in edible oil solution), a control group of head and neck skin amount of ethanol injection oil solvent. At 9:00 am on the 5th day of pregnancy, each group has random eight rats after anesthesia, extract the abdominal aortic blood, stripping of the uterine body. The rats were intraperitoneally injected with 3% sodium pentobarbital (0.2 ml/100 g), then the abdominal aorta of the rats was found, and the blood collection needle was inserted at an angle of 30 degrees toward the heart, and the depth of the needle was 5 mm. An 8- to 10-ml sterile blood could be obtained by vacuum blood collection. A portion of the fresh tissues was directly stored in cryovials at −80°C. Other tissues were put into 4% paraformaldehyde. Blood was collected from the rat’s abdominal aorta, and serum was obtained by centrifugation in serum separation tubes. Among the eight rats in each group, three rats were used as metabolomics samples, and the remaining five rats were used as experimental verification samples.

### Chemicals

High-performance liquid chromatography (HPLC) grade methanol and acetonitrile were purchased from Dikma Science and Technology Co. Ltd (Canada). HPLC grade formic acid was supplied by Beijing Reagent Company (Beijing, China).

### Plasma Preparation and Extraction

Aliquots of 100-μl frozen plasma samples were thawed and put into centrifuge tubes (1.5 ml), then they were thawed on ice, vortex for 10 s and mixed well, 300 μl of pure methanol was added to 50 μl of plasma/serum, whirl the mixture for 3 min and centrifuged it with 12,000 rpm at 4°C for 10 min. Then collect the supernatant and centrifuge it at 12,000 rpm at 4°C for 5 min. Leave in a refrigerator at −20°C for 30 min, centrifuge at 12,000 r/min at 4°C for 3 min and take 150 μl of supernatant in the liner of the corresponding injection bottle for on-board analysis.

### Metabolic Profiling by Ultraperformance Liquid Chromatography-MS

The sample extracts were analyzed using ultra-performance liquid chromatography coupled to a tandem mass spectrometry system (UPLC: Shim-pack UFLC SHIMADZU CBM30A, Kyoto, Japan; MS/MS: Applied Biosystems 4500 Q TRAP, Foster City, CA, USA). The liquid conditions were as follows: UPLC column, Waters ACQUITY UPLC HSS T3 C18 (1.8 μm, 2.1 × 100 mm); solvent system, water (0.04% acetic acid): acetonitrile (0.04% acetic acid); gradient program, 95:5 V/V at 0 min, 10:90 V/V at 10.0 min, 10:90 V/V at 11.0 min, 95:5 V/V at 11.1 min, 95:5 V/V at 14.0 min; flow rate, 0.40 ml/min; temperature, 40°C; injection volume, 5 μl. The effluent was alternatively connected to an ESI-triple quadrupole-linear ion trap (Q TRAP)-MS.

Linear ion trap (LIT) and triple quadrupole (QQQ) scans were acquired on a triple quadrupole-linear ion trap mass spectrometer (Q TRAP), API 4500 Q TRAP LC/MS/MS System, equipped with an ESI Turbo Ion-Spray interface, operating in a positive ion mode and controlled by Analyst 1.6.1 software (AB Sciex, Framingham, MA, USA). And the ESI source operation parameters were as follows: Ion source, turbo spray; source temperature 550°C; ion spray voltage 5500 V; curtain gas was set at 25.0 psi; the collision gas was high. Instrument tuning and mass calibration were performed with 10 and 100 μmol/L polypropylene glycol solutions in QQQ and LIT modes. QQQ scans were acquired as multiple reaction monitoring (MRM) experiments. Declustering potential (DP) and collision energy (CE) for individual MRM transitions were done with further DP and CE optimization [63]. According to the metabolites eluted within this period, a specific set of MRM transitions was monitored for each period according to the metabolites eluted.

### Metabolite Identification and KEGG Pathway Analysis

The VIP value determined significantly regulated metabolites between groups ≥ 1, fold change ≥ 1 (17). VIP values were extracted from OPLS-DA results, containing score plots and per-mutation plots generated using R package MetaboAnalystR. The data were log transform (log2) and mean centering before OPLS-DA to avoid overfitting.

Kyoto Encyclopedia of Genes and Genomes (KEGG, http://www.genome.jp/kegg/) pathway analysis is a database for determining the high-level functions and biological relevance of a large set of genes (18). Enrichment analysis was carried out for each metabolic pathway, and thermal map analysis of ploidy change was carried out for the enriched metabolic pathway. We selected the standard *P* value cutoff of 0.05 and performed the enrichment analysis. The RStudio 4.0.2 (ggplot2) was used to integrate the KEGG pathways (19).

### Experimental Validation

#### Obtaining Endometrial Tissue and Extracting Peripheral Blood

In each group, rats were anesthetized by the intraperitoneal injection of chloral hydrate (1:10 in physiologic saline) on day 5 of gestation. Half of the uterine tissue was taken from each rat in each group, and the uterus was cut lengthwise to expose the endometrium. After the number of pregnancies and embryo resorptions were counted, the tissues were rinsed with saline. For the abortion model, the placental deciduate tissues were obtained from fetal loss parts. A portion of the fresh tissues was directly stored in cryovials at −80°C as fresh tissue for real-time polymerase chain reaction (qRT-PCR) and western blot assay. Other tissues were put into 4% paraformaldehyde, and after 24 h, the tissues were embedded in paraffin used for HE. Blood was collected from the rat’s abdominal aorta, and serum was obtained by centrifugation in serum separation tubes. Serum was stored at −80°C and subsequently used in an enzyme-linked immunosorbent assay (ELISA).

#### Histopathological Examination (H&E Staining)

The endometrial tissues were fixed in 10% PBS neutral formalin, dehydrated, washed, and paraffin-embedded. The paraffin-embedded specimens were sectioned and stained with hematoxylin-eosin staining (H&E staining). In H&E staining endometrial tissues, the nucleic acids stain dark blue, and the proteins stain red to pink.

#### Immunohistochemical Assay (IHC)

Antigens were detected using the indirect method of enzyme immunohistochemistry. Half of the uteri were removed and fixed in paraformaldehyde (20). The uteri were removed, fixed in 4% paraformaldehyde in pH 7.0 phosphate-buffered saline (PBS) overnight and cryoprotected by immersion in 30% sucrose in PBS. Frontal sections of the uteri (15 μm thick) were cut using a microtome and mounted on gelatin-coated slides. Sections were treated with 0.01 M citrate buffer (pH 6.0) and heated in a microwave oven for 10 min at maximal power for antigen retrieval and rinsed in PBS for 15 min. Next, the tissue sections were treated with 3% H_2_O_2_ for 20 min to inhibit endogenous peroxidase activity and rinsed three times for 5 min in 0.01 M PBS (pH 7.2–7.4). To prevent non-specific binding, tissues were pre-incubated with normal rabbit serum, diluted 1:5 for 30 min at room temperature and then incubated with the COX-1 (1:150), COX-2 (1:500), PTGFR (1:300) and TBXA2R (1:500), GAPDH (1:500) antibodies, in a moist dark chamber overnight at 4°C. After the incubation with primary antibodies, the tissue sections were rinsed three times for 5 min in PBS. The sections were then incubated with HRP-labeled goat anti-rabbit IgG (H + L) (Beyotime, China) for 30 min at room temperature. A DAB horseradish peroxidase color development kit (Beyotime, China) was used for staining. Finally, all sections were examined under a microscope equipped with a digital camera system (Nikon, Tokyo, Japan). The cells with brown particles located at the cytoplasm of endometrial glandular epithelial cells, cavity epithelium cells, or stromal cells were considered positive staining cells. Three high-power fields were randomly selected under each high-power lens (×100) for each section, and there was no blank area in each field. The endometrial IHC staining section images were captured and measured using the image analysis software Image-Pro Plus 6.0. For each picture’s integrated optical density (IOD), each slice was randomly observed in 4 fields of view, and the IOD value of the endometrial protein was calculated. The average value was collected as the IOD value of the uterine specimen.

#### Western Blot

The BCA assay quantified proteins, and loading buffer 5X was added to the proteins, which were incubated for 5 min at 95°C. Then, proteins were loaded on an SDS-PAGE polyacrylamide gel, transferred to Immobilon-P PVDF membrane (Millipore, Shanghai, China), probed with the appropriate primary antibodies: COX-1 (1:5000), COX-2 (1:4000), and PTGFR (1:2000) were obtained from Abcam (Cambridge, UK), and TBXA2R (1:4000), GAPDH (1:10000), and detected by chemiluminescence (ECL, Thermo Scientific). Images were then acquired with Image-Lab software (Bio-Rad, Hercules, CA, USA). Image analysis of western blots was performed with Image-Lab analyzer software.

#### Reagents and Antibodies

COX-1 (ab109025), COX-2 (ab179800), and PTGFR (ab126709, ab188993) antibodies were obtained from Abcam (Cambridge, UK), and TBXA2R (27159-1-AP), GAPDH (10494-1-AP) antibodies were obtained from Proteintech (Chicago, USA).

#### Quantitative Real-Time Polymerase Chain Reaction (qRT-PCR)

According to the manufacturer’s instructions, total RNA was extracted from cell cultures using TRIzol^®^ (Life Technologies, Carlsbad, CA). RNA (1 µg) was reverse transcribed to cDNA templates using iScript cDNA synthesis kit (Bio-Rad, Hercules, CA). For semiquantitative RT-PCR, cDNA (25 ng) was amplified using SYBR^®^ Green PCR Master Mix (Life Technologies) and oligonucleotide primers for specific target sequences Applied Biosystems 7500 Real-Time PCR system. qRT-PCR parameters were as follows: denaturating at 95°C for 10 min, followed by 40 cycles of denaturing at 95°C for 15 s and annealing/extension at 60°C for 60 s. The system software automatically calculated threshold cycles (Ct). The expression of GAPDH normalized the expression level of the target mRNA. The collected data were quantified using the 2^−ΔΔCt^ method. Primer sequences are listed in **Table 1**.

**Table 1:** Primer sequences.

Name	Primer	Sequence
COX-1 COX-2 PTGFR TBXA2R GAPDH	Forward Reverse Forward Reverse Forward Reverse Forward Reverse Forward Reverse	5′-AACCGTGTGTGTGACTTGCTGAA-3′ 5′-AGAAAGAGCCCCTCAGAGCTCAGTG-3′ 5′-CACTCTATCACTGGCATCC-3′ 5′-TCTGCTC TGGTCAATGGA-3′ 5′-CGCTCAGTCCTCTGTTGTCG-3′ 5′-GCCACCCGATGTGAACTTTATG-3′ 5′-GAAGCAGACGGTTTGAGGGA-3′ 5′-TCAGTTTCCCCCGTGAATCG-3′ 5′-GATTTGGCCGTATCGGAC-3 5′-GAAGACGCCAGTAGACTC-3′

#### ELISA

Plasma supernatant was extracted and coated on a porous enzyme plate with a single antibody against rat Arachidonic Acid (E-EL-0051c), COX-2 (E-EL-R0792c), TBXA2R (E-EL-0057c), and PGF2α (E-EL-R0795c) all from (Elabscience, Wuhan, China). The Arachidonic Acid, COX-2, TBXA2R, and PGF2α in the samples and standards were combined with the single antibody, and the biotinized anti-rat antibody was added. Arachidonic acid, COX-2, TBXA2R, and PGF2α formed an immune complex that connected to the plate. Streptavidin labeled by horseradish peroxidase was combined with biotin, and the substrate working solution was blue. Finally, sulfuric acid was added to the termination solution, and the OD value was measured at 450 nm. The concentrations of Arachidonic Acid, COX-2, TBXA2R and PGF2α in the samples were directly proportional to the OD value, which could be calculated by drawing a standard curve.

### Statistical Analysis

All data are expressed as mean ± SD, obtained from more than three independent experiments, and analyzed by GraphPad Prism 9.0 (GraphPad Software, CA, USA). Statistically significant differences (*P < 0.05, **P < 0.01) were examined using the Student’s *t*-test and one-way ANOVA.

## Results

### Metabolomic Profiling of Plasma Samples and Multiple Statistical Analysis

**Figure 1:**
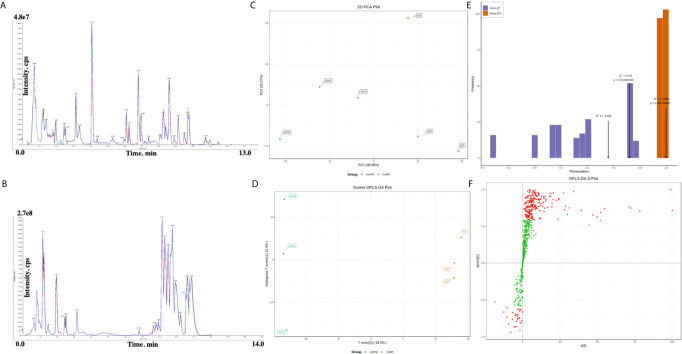
**(A, B)** Representative total ion chromatogram (TIC) obtained in electrospray ionization (ESI) positive ion mode from control group and RSA model group. **(C)** Principal components analysis (PCA) scores plot of the control group (green) and model group (red). **(D)** OPLS‐DA (Orthogonal partial least squares discriminant analysis) scores plot of the control group (green) and model group (red). **(E)** Validation plot obtained from permutation tests. **(F)** S‐plot of the OPLS‐DA model.

Representative UPLC-MS/MS total ion chromatograms (TICs) of the plasma from the two groups (**Figures 1A, B**). The relative standard deviation (RSD) values of the retention time and peak area of the QC sample were calculated positively and negatively. All of the results indicate that the repeatability and stability of the proposed method were statistically acceptable. This result confirms that the significant differences observed between the two groups using multivariate statistical analysis were more likely to result from genuine metabolites’ changes rather than from technical errors.

PCA and OPLS‐DA approaches are frequently used in metabolomics to classify groups suspected to show differences in metabolism. PCA, an unsupervised multivariate data analysis technique, was applied to assess the two groups’ trends in the initial cohort and potential outliers in the data. The scores plot showed no outliers in the data, and precise separation was achieved between the RSA group and the ion mode control group (**Figure 1C**). Then, we performed unsupervised OPCS‐DA to further differentiate the metabolite features and screen for potential marker metabolites. As shown by the scores plot (**Figure 1D**), the RSA group was separated from the normal controls, suggesting that metabolic perturbation had occurred in the RSA group. The OPLS‐DA analysis produced the following three key parameters: R^2^X, R^2^Y and Q^2^, which were 0.559, 0.999 and 0.721, respectively. When the metabolite data were acquired in positive and negative mode. Permutation testing (**Figure 1E**) was performed on the model’s quality and indicated that the model was not over‐fitted. The OPLS‐DA S‐plot (**Figure 1F**) can be used to show the differential metabolite profiles that are important for distinguishing the RSA group from the clustering of the two groups and may be regarded as potential biomarkers.

### Identification of Potential Biomarkers

**Figure 2:**
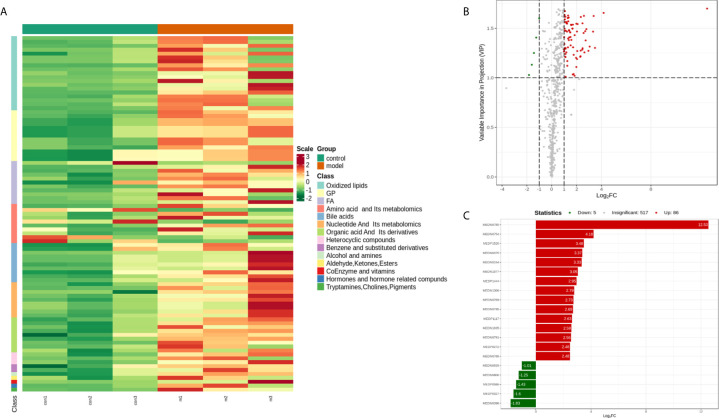
**(A)** Heat map analysis of 91 differential metabolites between control and model. The values of differential metabolites were normalized and shown as a color scale. The high and low metabolite levels were represented as reddish and greenish scales, respectively. **(B)** Volcano plot of differential metabolites between control and model. Red point, green point, and gray point indicate the metabolites that were significantly up-regulated, significantly down-regulated, and non-significantly different, respectively. **(C)** The bar plot of top 20 up-regulated metabolites and down-regulated metabolites.

**Figure 3:**
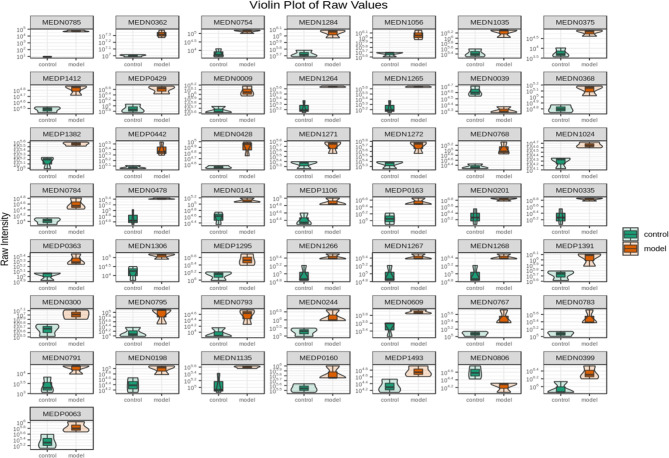
Violin plot analysis of 91 differential metabolites between control and model. The x axis indicates name of the groups, and the y axis indicates expression quantity.

To seek potential metabolites associated with RSA, we carefully selected several metabolites that contributed to its predictions. The VIP value ≥ 1, fold change ≥ 1 was selected the higher VIP score, the more reliable separation among the groups. 91 metabolites (Down: 5, Up: 86) are shown in **Table 2**. To more clearly characterize the plasma profile of RSA, we drew a heat map, differential metabolites violin diagram and the volcano plot based on the intensity levels of 91 markers between the two groups (**Figures 2**, **3**).

**Table 2:** Potential Biomarkers.

Formula	Biomarkers	VIP	Fold Change	Type	KEGG pathways
C4H7NO4 C10H17N3O6S C26H43NO5 C26H43NO5 C9H12N2O5 C6H8O7 C4H6O4 C5H4N2O4 C9H16O4 C4H6O4 C20H34O6 C20H32O3 C23H46NO7P C19H40NO7P C18H30O3 C20H30O2 C10H20O2 C20H40O2 C9H10O3 C14H17N5O8 C3H5NO4 C18H30O3 C7H13NO3S C13H14N2O3 C9H15N2O15P3 C20H32O3 C20H32O3 C22H32O3 C20H32O3 C18H32O3 C18H30O3 C22H32O3 C18H32O3 C18H30O3 C20H32O4 C20H34O5 C20H32O5 C20H34O5 C20H30O4 C7H12N2O4 C9H10O3 C6H11NO2 C18H32O3 C4H7NO4 C20H34O2 C22H35NO3 C24H40O5 C24H38O4 C24H38O4 C24H40O5 C19H40O3 C24H38O4 C27H48NO7P C27H48NO7P C27H46NO7P C27H46NO7P C27H46NO7P C25H48NO7P C25H48NO7P C23H42NO7P C5H8N2O5 C26H43NO5 C6H12N2O3 C6H9NO5 C10H13N5O4 C9H13N3O5 C10H9NO3 C13H16N2O4 C5H4N4O3 C9H11N5O3 C18H30O2 C8H20NO6P C11H15N5O5 C11H13NO3 C8H9NO C11H15N5O5 C24H40O3 C24H36O5 C9H13N3O5 C30H53NO7P C30H53NO7P C30H53NO7P C27H45NO4 C25H45NO4 C22H39NO6 C20H37NO4 C18H26O2 C22H30O2 C20H28O2 C11H15N5O4 C20H39NO4	l-Aspartica acid Glutathione reduced form Glycoursodeoxycholic acid Glycochenodeoxycholic acid 2’-Deoxyuridine Citric acid Succinic acid Orotic acid Azelaic acid Methylmalonic acid TXB2 [9α,11,15S-trihydroxythromba-5Z,13E-dien-1-oic (±)15-HETE [(±)15-hydroxy-5Z,8Z,11Z,13E Lysope 18:1 Lysope 14:0 13-HOTrE [13S-hydroxy-9Z,11E,15Z-octadecatrienoic EPA [5Z,8Z,11Z,14Z,17Z-eicosapentaenoic acid] Capric Acid(C10:0) Arachidic Acid(C20:0) 3-(3-Hydroxyphenyl)Propionate Acid N6-Succinyl Adenosine Aminomalonic Acid 9-HOTrE [9S-hydroxy-10E,12Z,15Z-octadecatrienoic N-Acetylmethionine Acetyl Tryptophan Uridine triphosphate (UTP) (±)12-HETE [(±)12-hydroxy-5Z,8Z,10E,14Z (±)16-HETE [(±)16-hydroxy-5Z,8Z,11Z,14Z (±)17-HDHA [(±)17-hydroxy-4Z,7Z,10Z,13Z,15E,19Z 11,12-EET [(±)11, (12)-epoxy-5Z,8Z,14Z-eicosatrienoic 12,13-EpOME [(±)12(13)epoxy-9Z-octadecenoic acid] 13-oxoODE [13-oxo-9Z,11E-octadecadienoic acid] 14(S)-HDHA [14S-hydroxy-4Z,7Z,10Z,12E,16Z,19Z 9,10-EpOME [(±)9,10-epoxy-12Z-octadecenoic acid] 9-oxoODE [9-oxo-10E,12Z-octadecadienoic acid] LTB4 [5S,12R-dihydroxy-6Z,8E,10E,14Z-eicosatetraenoic PGD1 [9α,15S-dihydroxy-11-oxo-prost-13E-en-1-oic acid] Prostaglandin E2 PGF2α [9α,11α,15S-trihydroxy-prosta-5Z,13E-dien-1-oic PGJ2 [11-oxo-15S-hydroxy-prosta-5Z,9,13E-trien-1-oic Nα-Acetyl-L-glutamine 3-(4-Hydroxyphenyl)-Propionic Acid D-piperidine acid 13(R)-HODE Iminodiacetic acid Homo-Gamma-Linolenic Acid N-arachidene glycine Alpha-Mercholic Acid 7-ketolithocholic acid 12-ketolithocholic acid Gamma-Mercholic Acid Heparin Orthocholic acid PysoPE 22:4(2n isomer1) PysoPE 22:4 PysoPE 22:5(2n isomer3) PysoPE 22:5(2n isomer2) PysoPE 22:5(2n isomer1) PysoPE 20:2(2n isomer1) PysoPE 20:2 PysoPE 18:3 N-Carbamoyl-L-aspartate Glycohyodeoxycholic acid D-Alanyl-D-Alanine N-Acetylaspartate Adenosine Cytidine 5-Hydroxyindole-3-Acetic Acid N-γ-Acetyl-N-2-Formyl-5-Methoxykynurenamine Uric acid Biopterin Punicic Acid Sn-Glycero-3-Phosphocholine 2-Methylguanosine N-Acetylphenylalanine 2-Phenylacetamide 7-methylguanosine Lithocholic acid 7,12-diketocholic acid Cytarabine LysoPC 22:5 (2n isomer3) LysoPC 22:5 (2n isomer2) LysoPC 22:5 (2n isomer1) Carnitine ph-C14 Carnitine C18:2 Carnitine C15:1:DC Carnitine C13:1 Octapentaenoic acid Docosaenoic acid Eicosahexaenoic acid N6-methyladenosine Carnitine C13:0 Isomer1	1.60 1.60 1.23 1.12 1.53 1.43 1.49 1.46 1.47 1.49 1.18 1.32 1.67 1.59 1.62 1.02 1.02 1.39 1.21 1.56 1.54 1.38 1.29 1.30 1.46 1.03 1.03 1.65 1.27 1.45 1.55 1.37 1.45 1.54 1.69 1.45 1.46 1.47 1.30 1.40 1.21 1.54 1.62 1.62 1.24 1.29 1.31 1.21 1.21 1.27 1.41 1.21 1.60 1.60 1.47 1.47 1.47 1.55 1.55 1.63 1.49 1.10 1.20 1.38 1.41 1.53 1.29 1.13 1.25 1.49 1.61 1.56 1.30 1.25 1.34 1.30 1.53 1.25 1.48 1.26 1.26 1.28 1.57 1.47 1.16 1.61 1.27 1.28 1.01 1.40 1.30	2.58 0.49 3.40 2.39 3.25 3.58 3.18 10.04 2.19 3.18 4.10 2.88 2.10 2.08 10.37 3.57 0.28 2.50 2.15 2.30 4.23 2.60 2.17 2.47 2.35 3.38 3.25 18.10 4.15 2.81 5.56 6.62 2.81 3.94 5929.92 5.91 4.08 6.47 3.29 0.42 2.15 2.36 6.02 2.50 2.15 2.13 8.29 4.97 4.97 5.02 2.85 4.90 2.46 2.46 2.64 2.64 2.64 2.30 2.30 2.05 6.89 3.94 3.88 3.00 2.00 2.98 5.59 0.32 2.27 2.05 5.06 2.14 2.15 0.37 2.03 2.15 2.9 6.19 2.63 2.00 2.00 2.00 2.53 2.06 2.39 2.15 7.74 5.51 2.17 2.66 11.18	Up Down Up Up Up Up Up Up Up up up up up up up up down up up up up up up up up up up up up up up up up up up up up up up down up up up up up up up up up up up up up up up up up up up up up up up up up up up down up up up up up down up up up up up up up up up up up up up up up up up	ko00220, ko00250 ko00270, ko00480 – ko00120, ko04976 ko00240, ko01100 ko00020, ko00250 ko00020, ko00190 ko00240, ko01100 – ko00240, ko00280 ko00590, ko01100 ko00590, ko04750 – – – ko01040 ko00061, ko01100 ko01040 ko00360, ko01100 – – – – – ko00240, ko01100 – – – – ko00591, ko01100 ko00591 – – ko00591 ko00590, ko01100 – ko00590, ko01100 – ko00590, ko01100 – – – – – – – – – – – ko04664, ko05133 – – – – – – – – – ko00240, ko00250 – ko00473, ko01100 ko00250, ko01100 ko00230, ko01100 ko00240, ko01100 ko00380, ko01100 ko00380 ko00230, ko01100 – – ko00564, ko00565 – ko00360, ko01100 ko00360, ko01100 – ko04976 – – – – – – – – – – – – – –

### Metabolic Pathway Analysis

**Figure 4:**
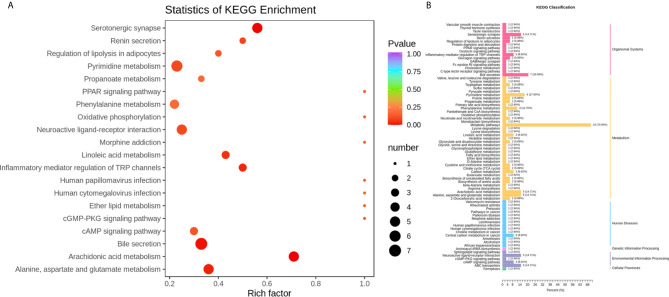
**(A)** Statistics of KEGG enrichment. The x axis indicates the rich factor corresponding to each pathway, and the y axis indicates name of the KEGG metabolic pathway. The size and color of bubbles represent the number and degree of enrichment of different metabolites, respectively. **(B)** KEGG classification of 74 pathways from 91 differential metabolites. The x axis indicates the proportion and number of metabolites annotated to the pathway, and the y Axis indicates name of pathway.

To identify the most relevant metabolic pathways involved in RSA, KEGG enrichment analysis was employed. Metabolic pathways involving Arachidonic acid metabolism, Serotonergic synapse, Bile secretion, Alanine, aspartate and glutamate metabolism, Inflammatory mediator regulation of TRP channels and Linoleic acid metabolism were highlighted as targets for investigating the pathological mechanisms underlying RSA (**Figure 4**). The *P*‐value and Corrected *P*-value threshold calculated from pathway topology analysis was set to 0.05 and 0.10, respectively. The Arachidonic acid metabolism (*P* = 0.0004, Corrected = 0.032) pathway was identified as a potential target pathway for RSA.

### Changes in Embryonic and Decidual Cell Morphology

**Figure 5:**
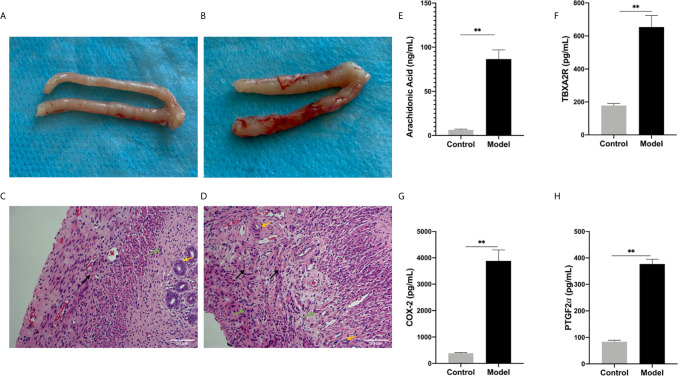
**(A, B)** Generally observing the uterus in every group. **(A)** Control group, **(B)** Model group. **(C, D)** The endometrium under optical microscope. **(C)** Blank control group, **(D)** model group. (Original magnification: × 200). The expression levels of various factors in serum: **(E)** Arachidonic Acid, **(F)** TBXA2R, **(G)** COX-2, **(H)** PGF2α. (**P < 0.01). Values are means ± SD, n = 3 per group.

In the control groups, the uterus had a pale pink appearance. In the model group, some of the embryos were almost completely absorbed as a non-pregnant state and the volumes of some embryos were smaller than those in the control groups. The fetal-placental unit had apparent congestion, which represented abortion embryos (**Figures 5A, B**). The endometrial were observed under an optical microscope after H&E staining. Decidual cells in the control groups had similar cytoplasmic staining with clear cell boundaries and tight packing. In contrast, in the model group, the endometrial stromal cells had degenerated and the connections between them had loosened. Moreover, some of endometrial stromal cells were necrotic and the nuclei had disappeared. When compared with the control group, there were thin endometria, sparse distribution of glands, proliferated interstitial cells, edema, proliferated and congested small blood vessels, and infiltrated inflammatory cells observed and some capillaries expansion and congestion in the model group (**Figures 5C, D**).

### Arachidonic Acid Metabolism Involved in The Pathogenesis of RSA

**Figure 6:**
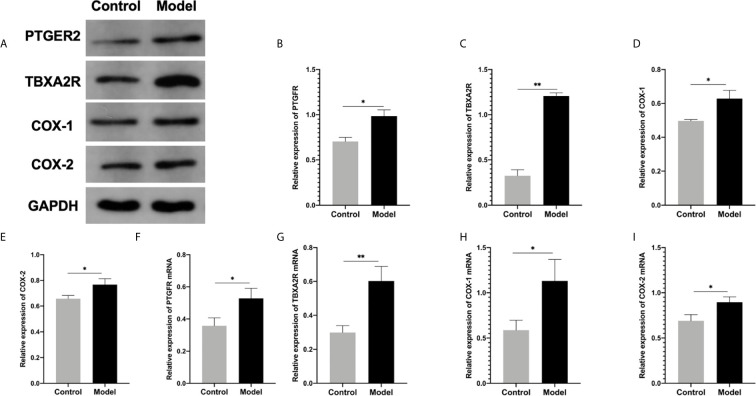
**(A–E)** Western blot for the protein expression in endometrial tissue in rats: **(B)** PTGFR, **(C)** TBXA2R, **(D)** COX-1, **(E)** COX-2: (*P < 0.05 **P < 0.01). mRNA expression in endometrial tissue in qRT-PCR: **(F)** PTGFR, **(G)** TBXA2R, **(H)** COX-1, **(I)** COX-2: (*P < 0.05 **P < 0.01). Values are means ± SD, n = 3 per group.

**Figure 7:**
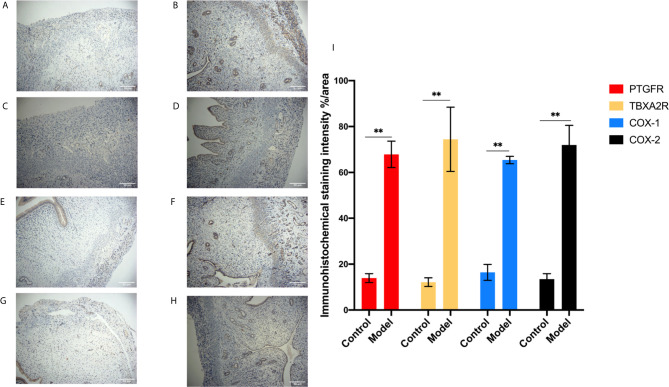
IHC for the protein expression in endometrial tissue in rats. **(A, B)** The expression of PTGFR: control **(A)**, model **(B)**. **(C, D)** The expression of TBXA2R: control **(C)**, model **(D)**. **(E, F)** The expression of COX-1: control **(E)**, model **(F)**. **(G, H)** The expression of COX-2: control **(G)**, model **(H)**. **(I)** % area for the immunohistochemical staining intensity in endometrial tissue in rats. Bars with different letters are statistically different (**P < 0.01). Values are means ± SD, n = 3 per group.

To investigate the role of the Arachidonic acid metabolism of proteins in RSA regulation, we examined the mRNA and protein levels of COX-1, COX-2, TBXA2R, and PTGFR in the two groups’ uterus. The TBXA2R and PTGFR mRNA and protein expression levels showed a gradual increase in the model group (*P* < 0.05) (**Figure 6**). Furthermore, we examined COX-1, COX-2 expression in RSA *in vitro*. The results showed that RSA induces COX-1 and COX-2 mRNA increased, and their protein expression was higher in the model group (*P* < 0.05) (**Figures 6H, I**). In Elisa assay (**Figures 5E–H**), the serum Arachidonic Acid levels in the control group were significantly lower than those in the RSA group. In contrast, the serum COX-1, COX-2, TBXA2R, and PTGFR levels were all higher after abortion induction than the control group (*P* < 0.05). Meanwhile, in the IHC assay, the model group significantly increased the COX-1, COX-2 expression in the endometrial glandular epithelium (*P* < 0.05) compared with the control group. The TBXA2R and PTGFR expression in the endometrial glandular epithelium in model rats was significantly increased when compared to the normal controls (*P* < 0.05) (**Figure 7**).

## Discussion

The changes of serum metabolite profiles between the RSA rat model and normal control group were studied. UPLC-MS/MS was used for comprehensive metabolomics analysis to provide the overall metabolite changes. Multivariate statistics analyzed the recurrent abortion model group and the control group. From the PCA load map, potential biomarkers were shown as positive and negative ion patterns, separating the model from the control group. Independent *P*-values showed significant differences in 91 metabolites and 74 KEGG pathways between the two groups. Arachidonic acid metabolism is the most influential, so we chose this pathway as our experimental verification target in the subsequent experiments.

Metabolomics is an essential branch in genomics research (21), and it is also a high-throughput and impartial analysis technique for the study of metabolic pathways *in vivo*. Metabolomics can reflect the overall changes of early metabolites after high-throughput metabolic analysis (22, 23). An essential component of systems biology complements the limitations of genomics, transcriptomics, and proteomics. It is a means of measuring the metabolic profile of small molecules and the flux after genetic modification and exogenous challenges in the biological stroma. Due to the biological is the final product of cell metabolism, compared with other “omics” technology, use of metabolites are the benefits of omics, produced by the former data is likely to reflect current epigenome, genome, and proteome or transcriptome level any tiny physiological changes, it can be an insight into the overall metabolic state of the organism.

At least 25% and up to 50% of women experience one or more recurrent miscarriages (24), and this risk increases with the age of the pregnant woman. However, currently commonly used drugs cannot achieve the desired therapeutic effect. Therefore, it is of great significance to seek more effective prevention and control methods and improve the therapeutic effect of RSA.

Arachidonic acid (AA) plays an important role in female reproduction and abortion. AA is metabolized to prostaglandin F2α (PGF2α) in utero and is involved in various reproductive activities such as luteinolysis, maternal pregnancy recognition, endometrial gene expression, and development (25–27). Therefore, the synthesis of prostaglandin depends on the detectability of AA (28) and the activity of enzymes involved in its metabolism (29). Prostaglandins, produced by the cyclooxygenase of arachidonic acid, stimulate uterine contractions. Both PGF2α and TXA2 were significantly correlated with AA, and the levels of AA, PGF2α, PGE2, and TXA2 in the amniotic fluid of patients with abortion or delivery were significantly increased (30). Free AA was released during abortion and delivery, leading to increased synthesis of PGF2α, PGE2-prostacyclin, and thromboxane A2 (TXA2) in the membranes and decuviae (31), which may be related to the process of abortion and delivery in patients. AA metabolites regulate uterine contractions (32). Although maintaining an inflammatory state during pregnancy allows the mother to tolerate the fetus, overactivation of inflammation may lead to pathological pregnancy, such as premature delivery and miscarriage (33). We detected that the serum AA level of the RSA model rats was higher than that of the control group, which may be caused by the significant increase of free arachidonic acid and total arachidonic acid in the serum of women caused by early pregnancy abortion.

Prostaglandin is one of the critical media of the female reproductive system, it involved in ovulation, fertilization, and pregnancy (34) maintenance or delivery process, possible due to the increase of free arachidonic acid release or remove H prostaglandin synthase activity restrictions, or both synergy results (35). However, there was still a large amount of PGF2α in the rats’ uterus with repeated abortion, and the same results were found at mRNA level, which may be related to a large amount of arachidonic acid in the serum of the model rats. PGF2α plays a biological role through the prostaglandin F2α receptor (PTGFR), a G-protein-coupled receptor. Next, we analyzed the presence of functional regulation of PGF2α and PTGFR in RSA rats. Not only PGF2α but also PTGFR increased significantly in the uterus of RSA rats, which combined and interacted with each other to contract the uterus and lead to abortion. Prostaglandins are bioactive lipids known to be the primary mediators of pathological conditions and are also essential in the female reproductive process (36). Our results are similar to those of previous studies: in dairy cows, increased uterine PGF2α concentrations are associated with higher embryonic mortality and reduced pregnancy rates (37, 38). In addition, the addition of PGF2α in the medium decreased *in vitro* development of rabbit (39) and rat (40) embryos and *in vitro* and *in vivo* development of bovine embryos (41).

AA regulates the cytoplasmic phospholipase A2α/cyctoxase-2 (COX-2) pathway in endometrial stromal cells (42) and induces prostaglandin synthesis, which plays a vital role in embryo implantation and decarboxylation (43). Two subtypes of COX enzyme (COX-1 and COX-2) were distinguished. COX-1 is involved in critical physiological functions, such as control of platelet aggregation, while COX-2 is mainly involved in inflammation and pathophysiological processes (44). Because of the crucial role of COX in uterine contraction, we investigated its expression in RSA rats. Interestingly, we found the same results in Western Bolt and IHC, with COX-1 and COX-2 overexpression in RSA model rats’ uterine tissue compared to the control group. Therefore, we further verified the results at the mRNA level, which was consistent with the previous results, suggesting that AA-mediated COX-1 and COX-2 are involved in the occurrence and development of RSA. Through specific cox-2 gene knockout technology and animal embryo implantation, especially III period and closely related implantation, it is considered a critical factor of the mammalian embryo implantation (45). COX-2 is a rate-limiting enzyme that catalyzed the conversion of arachidonic acid to prostaglandin in the human body. It is an important messenger and effector molecule in the human body. Cox-2 expression in villi and decidua tissue may play a role in inhibiting apoptosis (46). Still, when under various cell cox-2 can rapidly, leading to increased prostaglandin synthesis, ultimately is not conducive to continue to pregnancy, prompt COX for normal pregnancy embryo implantation plays a vital role in regulating, COX embryo mother interface signal transduction pathway is of great significance for early pregnancy. The up-regulation of COX-2 can also up-regulate various inflammatory mediators and initiate multiple signaling pathways (47). Such a vicious cycle leads to the increased expression of COX-2 in the decuvium cells, which may lead to vasculoinflammatory response, intravascular coagulation cascade reaction, cell membrane damage, and cell apoptosis, and may lead to abortion in early pregnancy (48, 49).

In addition, TXA2 is an unstable arachidonic acid metabolite, a metabolite of arachidonic acid conversion thromboxane, which promotes platelet aggregation, procoagulant, and vasoconstriction. TXA2 and its receptor Thromboxane A2 receptor (TBXA2R) affect the transfer of placental nutrients from the mother to the fetus by regulating vascular tone (50, 51). Our study also showed that TBXA2R expression was increased in the model group but not in the other groups. The study measured urinary excretion of prostacyclin metabolites and thrombotic A2 metabolites. These results suggest that activation of TXA2 and TBXA2R may be a factor in recurrent abortion (52). It maintains a dynamic balance with prostaglandin in the body, which plays a vital role in maintaining vascular tension and preventing platelet aggregation. Once the balance of TXA2 and prostaglandin is broken, thrombus or bleeding tendency can be caused. Studies have found that the imbalance of the TXA2/PGF1α system *in vivo* after medical abortion is one of the mechanisms leading to bleeding after an abortion. TXA2 promotes the release of 5-HT, ADP, etc., and further enhances vascular inflammation and platelet aggregation (53). Thrombosis causes pathological changes in the placenta, damages the fetus-placenta unit’s function, and causes ischemia, hypoxia, and death of the embryo and fetus, resulting in abortion (54, 55). This experimental study also has some limitations. It was only verified in the samples of the rat model. In the future, we will carry out further screening and verification of clinical samples and cell samples, so as to improve the whole design and provide experimental basis for the treatment of RSA.

## Conclusion

In conclusion, in the present study, the UPLC-MS/MS based metabolomics method was applied to analyze the metabolic profile changes between RSA rats and controls. The identification of 91 potential biomarkers has shown that the RSA is closely related to AA metabolism and may be further investigated for diagnosis and therapy. Experimental verification results show that the AA pathway does play a vital role in the development of RSA. Aberrant the genes expression of COXs, TBXA2R and PGF2α in RSA rats’ uterine contribute to uterine contraction and ultimately to RSA. Several factors interact with each other, and this interaction leads to the overexpression of AA metabolism. Therefore, AA metabolism has the potential to be a useful therapeutic target in the treatment of RSA.

## Data Availability Statement

The original contributions presented in the study are included in the article/supplementary material. Further inquiries can be directed to the corresponding authors.

## Ethics Statement

The animal study was reviewed and approved by the Institutional Animal Care and Use Committees of Xi’an Jiaotong University.

## Author Contributions

All authors read and edited several draft versions and all approved the final manuscript. ML and YH: data curation and writing—original draft preparation. MK and PA: methodology and software. XW: conceptualization. HD: visualization and investigation. XX: supervision and writing—reviewing and editing.

## Funding

This work is supported financially by the National Natural Science Foundation of China (NSFC) No. 81703797, the Major Research Development Program of Shaanxi No. 2020SF-278 and State Administration of Traditional Chinese Medicine Liu Runxia Famous Old Traditional Chinese Medicine Studio.

## Conflict of Interest

The authors declare that the research was conducted in the absence of any commercial or financial relationships that could be construed as a potential conflict of interest.

## Abbreviations

AA, arachidonic acid; CE, collision energy; COX-2, cytosolic phospholipase A 2α/cyclooxygenase 2; COX-1, cytosolic phospholipase A 2α/cyclooxygenase 1; DP, declustering potential; ELISA, enzyme-linked immunosorbent assay; HPLC, high performance liquid chromatography; H&E, histopathological examination; IHC, immunohistochemical assay; IOD, integrated optical density; KEGG, Kyoto Encyclopedia of Genes and Genomes; LIT, linear ion trap; MRM. multiple reaction monitoring; OPLS‐DA, orthogonal partial least-squares discriminant analysis; PBS, phosphate-buffered saline; PCA, principal component analysis; PGF2α, prostaglandin F2 alpha; PTGFR, prostaglandin F2α receptor; qRT-PCR, real-time polymerase chain reaction; QQQ, triple quadrupole; RSA, recurrent spontaneous abortion; RSD, relative standard deviation; TXA2, metabolizes AA to produce thromboxane A2; TBXA2R, thromboxane A2 receptor; TICs, total ion chromatograms; UPLC, ultra-performance liquid chromatography.
